# Supplementary material for: Development of the BioHybrid Assay: Combining Primary Human Vascular Smooth Muscle Cells and Blood to Measure Vascular Calcification Propensity
Source: Cells. 2021 Aug 16;10(8):2097. doi: 10.3390/cells10082097 (PMC8391733; doi:10.3390/cells10082097)
Supplement: Supplementary file 1 [file cells-10-02097-s001.zip › cells-1313675-supplementary.pdf]

# SUPPLEMENTAL

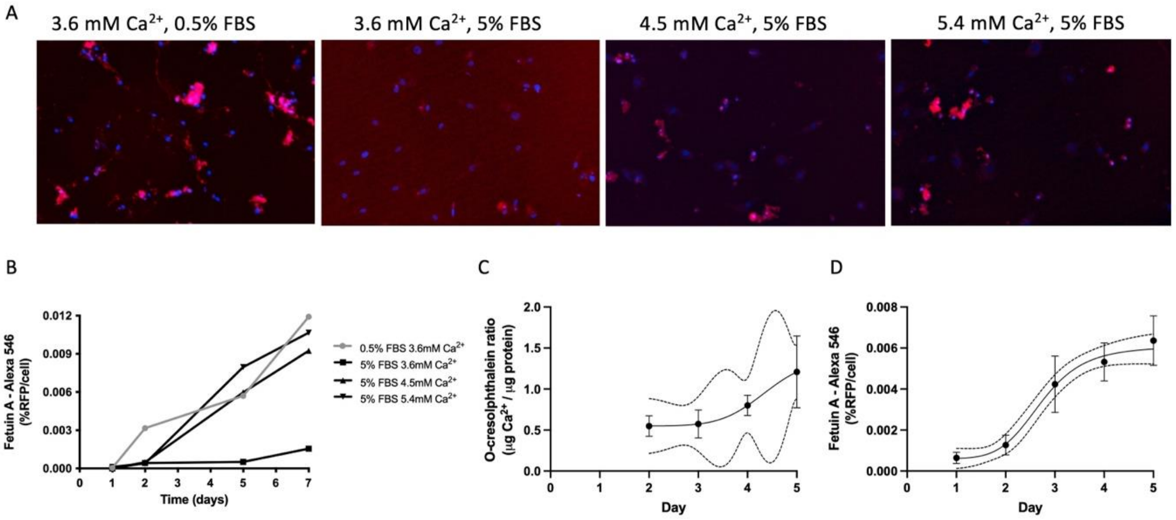


**Figure S1.** Validation of the BioHybrid assay. (A) Representative images of hVSMC calcification in the BioHybrid assay under different calcium concentrations. (B) RFP signal over time, to determine the best calcifying conditions which are not similar to the 0.5% serum condition (positive control). (C) Sigmoidal interpolation of o-Cresolphthalein assay. (D) Sigmoidal interpolation of BioHybrid assay.

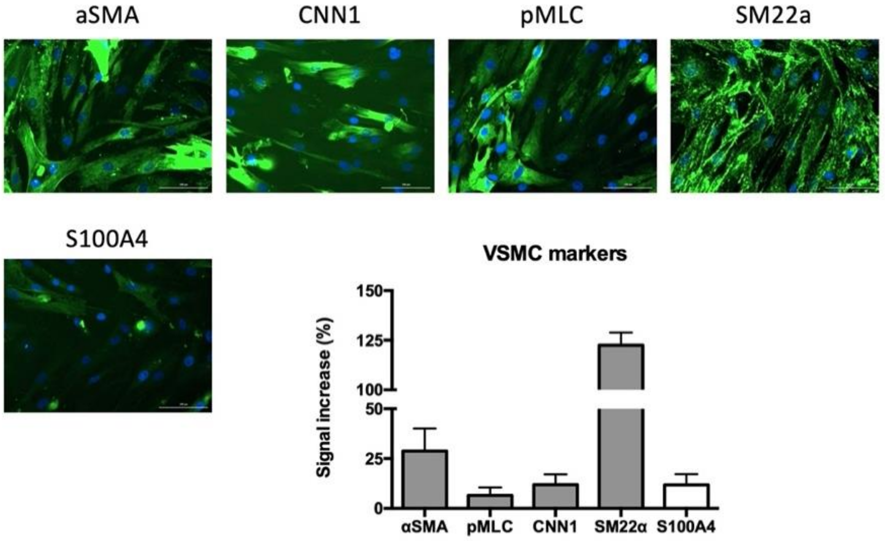


**Figure S2.** Characterisation of hVSMC. Immunocytochemical images on contractile (alpha Smooth Muscle Actin (αSMA), Calponin (CNN1), phosphorylated Myosin Light Chain (pMLC) and Smooth Muscle protein 22 alpha (SM22α)) and synthetic (S100 calcium binding protein A4 (S100A4)) hVSMC markers. Lower right panel: signal increase of markers compared to background.
